# Personalized alignment in total knee arthroplasty reduces patellar tilt: A retrospective study

**DOI:** 10.1002/ksa.70097

**Published:** 2025-10-17

**Authors:** Alexandre Le Guen, Simon Marmor, Vincent Le Strat, Antoine Mouton, Thomas Aubert

**Affiliations:** ^1^ Department of Orthopaedic Surgery Groupe Hospitalier Diaconesses Croix Saint‐Simon Paris France

**Keywords:** patellar tilt, personalized alignment, robotic assistance, total knee arthroplasty

## Abstract

**Purpose:**

Patellofemoral kinematics is crucial for patient satisfaction following total knee arthroplasty (TKA). A postoperative patellar tilt greater than 5° has been associated with inferior clinical outcomes. While a femoral component external rotation beyond 3° is generally protective against patellar tilt, personalized alignment may not consistently reach this threshold. The aim of this study is to (1) assess the incidence of postoperative patellar tilt >5° following TKA with personalized alignment and (2) identify preoperative and intraoperative factors associated with this complication.

**Methods:**

This comparative retrospective study included 316 primary TKAs Attune (Johnson & Johnson®) performed between January and December 2024 using navigation or robotic assistance. All procedures were conducted at the same center by four surgeons: two using a subvastus approach and two a transquadricipital approach. Thirty‐six involving residents were excluded. Patellar tilt is defined as an angle exceeding 5° between a line drawn along the anterior borders of the femoral condyles and a line connecting the posterior edges of the medial and lateral patellar facets on a patellofemoral skyline view 3 months postoperatively. No patients were lost to follow‐up. To identify postoperative patellar risk factors, the following data were analyzed: Age, gender, body mass index, preoperative hip‐knee‐ankle angle, medial proximal tibial angle, lateral distal femoral angle, coronal plane alignment, functional knee phenotype, tibial slope, preoperative radiographic patellar tilt, range of motion, surgical approach, laterality, implant type (posterior‐stabilized or cruciate‐retaining), use of robotics or navigation, patellar resurfacing and patellar button size (if applicable).

**Results:**

Patellar tilt was observed in 11.8% of cases, a rate markedly lower than that reported for mechanical alignment in existing literature. Multivariate analysis identified the transquadricipital approach (odds ratio [OR]: 2.75; *p* = 0.02), right‐sided surgery (OR: 2.63; *p* = 0.02) and PS implants (OR: 2.59; *p* = 0.04) as independent risk factors.

**Conclusion:**

Personalized alignment, the subvastus approach, and cruciate‐retaining implants appear to reduce the risk of postoperative patellar tilt.

**Level of Evidence:**

Level III, retrospective cohort study.

**Abbreviations**
CPAK: coronal plan alignment knee
CR: cruciate retaining
FKP: functional knee phenotype
PS: posterior stabilized
TKA: total knee arthroplasty

## INTRODUCTION

Postoperative anterior knee pain and patellar complications remain common after total knee arthroplasty (TKA) [13, 21]. Patellar maltracking is the leading patellofemoral issue, often causing pain and limited motion [22, 25, 44]. Despite improvements in implants and techniques, these complications still account for 10% of all TKA issues and about 45% of early revisions, regardless of patellar resurfacing [2, 30, 37, 47].

One way to evaluate patellofemoral complications is by assessing postoperative patellar tilt which has been associated with inferior clinical outcomes [12, 36]. It is defined as an angle greater than 5 degrees between a line drawn along the anterior borders of the femoral condyles and a line connecting the posterior edges of the medial and lateral patellar facets [19]. Femoral component rotation directly influences knee kinematics by affecting its stability in flexion and the patellofemoral joint tracking [35, 45]. In mechanical alignment, femoral component external rotation exceeds 3° to prevent patellar tilt, while personalized alignment focuses on ligament balance and may not reach this threshold [8, 21, 34].

To date, few studies exist on the impact of personalized alignment on the incidence of post operative patellar tilt [21, 24]. Existing studies do not consider the potential impact of emerging technologies, such as robotic assistance, which may enhance precision and optimize the accuracy of implant positioning and therefore could have an impact on patellofemoral kinematics [9, 43].

The main objective of this study was to assess the rate of postoperative patellar tilt greater than 5 degrees at 3 months postoperatively when using personalized alignment. The secondary objective was to identify risk factors using pre‐ and intraoperative collected data. Our hypothesis was that patient‐specific alignment does not lead to increased patellar tilt compared to the rates reported in existing literature on mechanical alignment.

## MATERIALS AND METHODS

### Study design

This study is a retrospective analysis of a single center consecutive patient series. Inclusion criteria were as follows: primary TKAs Attune (Johnson & Johnson®, Medtech), with either VELYS robotic‐assisted system or navigated assistance (Brainlab Knee3 Navigation), from January 2024 to December 2024 in the same department. All procedures were performed by four surgeons, two employing a subvastus approach and two a transquadricipital approach. A cemented, fixed‐bearing primary total knee implant was used in all cases with no tourniquet applied during surgery. Patients undergoing unicompartmental arthroplasties or TKA revisions were not eligible for inclusion in this study. The exclusion criteria were as follows: cases where the procedure was partially performed by a resident.

**Figure 1:**
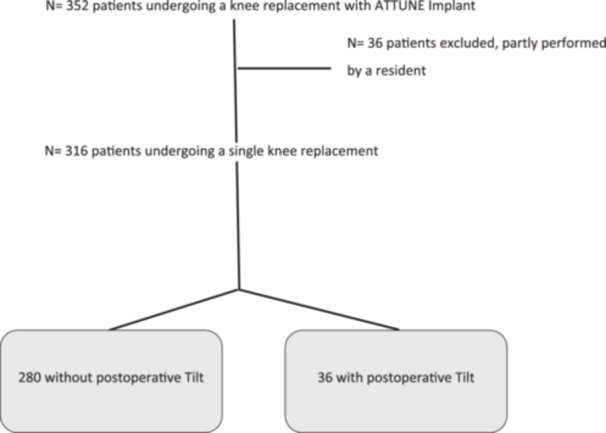
Flow diagram of patient selection for the analyses.

Of the 352 eligible patients, 36 were excluded as their procedures were partially performed by a resident. Of the 316 patients included, 133 were operated using the VELYS™ Robotic‐Assisted Solution (Johnson & Johnson, Medtech) and 183 using the navigated instrumentation (Brainlab Knee3 Navigation). At 3 months postoperatively, patients were classified into two groups based on the presence of patellar tilt on the axial radiograph: group with no patellar tilt (*n* = 280) and group with patellar tilt (*n* = 36) (Figure 1)**.** Three months postsurgery, not a single patient has been lost to follow‐up. Patient demographics are shown in Table 1.

**Table 1:** Baseline characteristics of patient.

	**Tilt** < **5°**	**Tilt** ≥ **5°**	***p*‐Value**
	*n* = 280	*n* = 36	
Baseline characteristic			
Age (years), mean (range)	73.5 (50–93)	74.5 (66–85)	0.345
Male sex, no. (%)	93 (33.2%)	12 (33.3%)	>0.999
Body mass index, km/m^2^ (range)	287.7 (17.6–38.1)	28.8 (22–36.3)	0.366
Surgical parameter			
Side right, mean (%)	140 (50%)	27 (75%)	**0.008**
Hip‐knee‐ankle initial (°), mean (range)	175.7 (159–206)	174.9 (163–195)	0.426
Recurvatum (°), mean (range)	0.1 (0/10)	0 (0/0)	0.422
Flexion contracture (°), mean (range)	3.1 (0/30)	2.6 (0/15)	0.731
Flexion (°), mean (range)	110 (60/140)	112 (80/140)	0.691
Approach sub/mid vastus, no (%)	190 (67.9%)	12(33.3%)	**<0.001**
Cruciate retaining (vs. posterior stabilized), no (%)	172 (61.4%)	10 (27.8%)	**<0.001**
Use of robot (vs. navigation)	124 (44.3%)	9 (25%)	**0.043**
Patellar resection asymmetry > 2 mm, *n* (%)	53 (21.1%)	7 (20.6%)	>0.999
Patellar resurfacing, *n* (%)	249 (88.9%)	34 (94.4)	0.398
Preoperative Tilt (°), mean (range)	3.1 (0.0–19.0)	3.6 (0.1–18.3)	0.866
Preoperative medial proximal tibial angle (°), mean (range)	87.1 (74.0–98.0)	86.9 (80.0–95.0)	0.718
Preoperative lateral distal femoral angle (°), mean (range)	89.4 (74.0–99.0)	88.7 (84.0–94.0)	0.16
Preoperative tibial slope (°), mean (range)	5.2 (0.0–20.0)	5.2 (1.0–10.0)	0.962

Before surgery, all patients underwent a clinical examination by a knee surgeon and had a knee X‐ray consistent with a diagnosis of symptomatic knee osteoarthritis. Limb alignment (hip‐knee‐ankle [HKA] angle), preoperative medial proximal tibial angle (MPTA), lateral distal femoral angle (LDFA), coronal plan alignment knee (CPAK), functional knee phenotype (FKP) and tibial slope were measured on full length X‐rays based on a standardized protocol [15, 17, 29, 42]. Preoperative radiographic patellar tilt was measured on a patellofemoral skyline view. Measurements were conducted using the MediCAD software system (mediCAD, Hectec GmbH) and were repeated twice, 1 month apart, by a senior surgeon. The reliability and accuracy of this method, including both inter‐ and intraobserver agreements, has been reported in relevant literature as good to excellent [5, 23].

The preoperative clinical examination included a standard knee examination with the knee range of motion (Recurvatum‐Flexion contracture‐Maximal flexion). The following clinical information was collected: age, gender, body mass index and American Society of Anesthesiologists Score [39].

Approach (transquadricipital or subvastus), size of implants including patellar button size (if applicable), and constraint of the prosthesis (cruciate retaining or posterior stabilized) were recorded. Patellar resurfacing was not systematically performed by two of the four surgeons. Surgeons performing selective patellar resurfacing did so in cases of advanced patellofemoral osteoarthritis (Iwano grade 4). However, resurfacing was avoided in cases where the patella was excessively thin (less than 9 mm) to avoid the risk of fracture.

**Figure 2:**
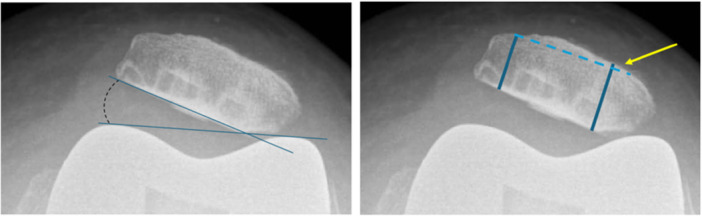
Radiological measurements of patellar tilt and asymmetry.

All patients had a postoperative radiographic assessment at 3 months which included: anteroposterior view, lateral view, weight bearing view, patellar axial view. Axial views were performed using the Merchant method [33]. Patellar tilt was defined as an angle exceeding 5° between a line drawn along the anterior borders of the femoral condyles and a line connecting the posterior edges of the medial and lateral patellar facets, measured on a patellofemoral skyline view 3 months postoperatively (Figure 2) [12, 19].

Patellar resection asymmetry was assessed as the difference between the thickest and the thinnest measurements of the residual patella after resection. The patellar asymmetry resection threshold was 2 mm (Figure 2) [1, 32].

This study was approved by the local institutional review board *(CNIL MR004 2225508)* and complies with the principles of the Helsinki declaration.

### Personalized alignment philosophy [26]

All four surgeons followed the same personalized alignment philosophy using a tibia‐first, gap‐balanced technique. Implant positioning aimed to restore each patient's native soft tissue laxity, thereby avoiding ligament releases [10, 16, 48].

Deformities measuring less than 10° were corrected to a mechanical axis between 0° and 3°, while severe deformities (>10°) were corrected to a range between 0° and 5°. Preoperative varus knees were intentionally corrected to a postoperative HKA angle of less than 180°, while valgus knees were adjusted to remain within a targeted safe zone, with HKA angles ranging from 177° to 183° [11].

First, medio‐lateral balancing in extension is targeted using a tibial cut obliquity between 3° of valgus and 3° of varus and the distal femoral cut obliquity ranged from 5° of valgus to 5° of varus, depending on the coronal plane deformity. At full knee extension, medial‐lateral laxity differences were targeted to be <1 mm [4].

Then, medio‐lateral balancing in flexion is managed by the obliquity of the posterior femoral cut, which modifies the rotation of the femoral component. This rotation was restricted between 6° of external rotation and 2° of internal rotation. At 90° flexion, medial‐lateral laxity differences were kept below 2 mm [4].

Balancing of the extension–flexion spaces is managed by adjusting the thickness of the tibial or distal femoral cuts, the flexion of the femoral component, or its anteroposterior positioning. From full extension to 90° of flexion, the medial laxity difference aimed to be <1 mm [4].

The tibial cut can be increased in cases of significant tibial wear or decreased in cases of excessive resection (9 mm on the less used compartment), or in cases of genu valgum (under‐resected to 7 mm).

The thickness variation of the distal femoral cut was restricted to ±2 mm to minimize patellofemoral stress during flexion and mid‐flexion instability.

Finally, a femoral flexion component between 3° and 6° allows us to reduce the flexion gap and the risk of notching. The anteroposterior positioning of the femoral implant was optimized to fine‐tune the flexion gap while avoiding anterior overhang and posterior notching.

### Statistical analyses

Before analysis, missing or inconsistent data were verified and corrected, after which the database was locked. Patient characteristics were summarized using descriptive statistics: mean (SD) for continuous variables and frequency (%) for categorical variables. Group comparisons were made using the *χ*²‐test or Fisher's exact test for categorical data, and Student's *t*‐test or Mann–Whitney test for continuous data, depending on distribution. Factors independently associated with postoperative tilt were identified using logistic regression, including variables significant at *p* < 0.20 in univariate analysis. The final model was obtained by stepwise regression, with model fit assessed via likelihood ratio test. All *p*‐values were two‐sided, with significance set at *p* < 0.05. Statistical analysis was performed using EasyMedStat Version 3.40.

## RESULTS

A total of 316 patients were included in the study, of whom 122 (38.6%) were men. The median age at the time of surgery was 74 years (range, 50–93). Patellar resurfacing was performed in 283 patients (89.5%).

The overall postoperative incidence of patellar tilt was 11.8% across the cohort. Patient characteristics, stratified by the presence or absence of postoperative patellar tilt, are summarized in Table 1.

The CPAK distribution of patients was as follows: type 1 in 35.4% of cases (112 patients), type 2 in 18.7% (59), type 4 in 15.2% (48), type 3 in 13.3% (42), type 5 in 12.7% (40), type 6 in 4.4% (14), type 0 in 0.3% (1), and no patients were classified as CPAK type 7 or 8. The main HKA, femoral and tibial phenotypes were VAR_HKA_9° (59 patients, 18,9%), NEU_FMA_0° (133 patients, 42,6%) and NEU_TMA_0° (134 patients, 42,9%). The most common phenotype was VAR_HKA_9° + VAR_FMA_3° + NEU_TMA_0° (23 patients, 7,4%). The main phenotypes by gender [6] are presented in Table 2.

**Table 2:** The 10 most common functional knee phenotypes of the population.

Rang	*N*	%	Functional knee phenotype
Males			
1	14	6.7	VAR_HKA_9°NEU_FMA_0°VAR_TMA_3°
2	13	6.2	VAR_HKA_9°VAR_FMA_3° NEU_TMA_0°
3	13	6.2	VAR_HKA_6°VAR_FMA_3° NEU_TMA_0°
4	9	4.3	VAR_HKA_12°VAR_FMA_3° NEU_TMA_0°
5	7	3.3	VAR_HKA_3°NEU_FMA_0° NEU_TMA_0°
6	7	3.3	VAR_HKA_6°NEU_FMA_0°VAR_TMA_3°
7	6	2.9	VAR_HKA_12°NEU_FMA_0°VAR_TMA_3°
8	6	2.9	VAR_HKA_6°NEU_FMA_0° NEU_TMA_0°
9	5	2.4	VAL_HKA_6°NEU_FMA_0°VAL_TMA_3°
10	4	1.9	VAR_HKA_6°VAR_FMA_3°VAL_TMA_3°
Total	84	40.1	
Females			
1	10	9.7	VAR_HKA_9°VAR_FMA_3° NEU_TMA_0°
2	8	7.8	VAR_HKA_6°VAR_FMA_3° NEU_TMA_0°
3	7	6.8	VAR_HKA_12°VAR_FMA_3°VAR_TMA_3°
4	7	6.8	VAR_HKA_3°VAR_FMA_3° NEU_TMA_0°
5	5	4.9	VAR_HKA_9°VAR_FMA_3°VAR_TMA_3°
6	4	3.9	VAR_HKA_3° NEU_FMA_0°VAR_TMA_3°
7	4	3.9	VAR_HKA_9° NEU_FMA_0°VAR_TMA_3°
8	3	2.9	VAR_HKA_12°VAR_FMA_3° NEU_TMA_0°
9	3	2.9	VAR_HKA_15° NEU_FMA_0°VAR_TMA_3°
10	3	2.9	VAR_HKA_3° NEU_FMA_0° NEU_TMA_0°
Total	54	52.5	

**Figure 3:**
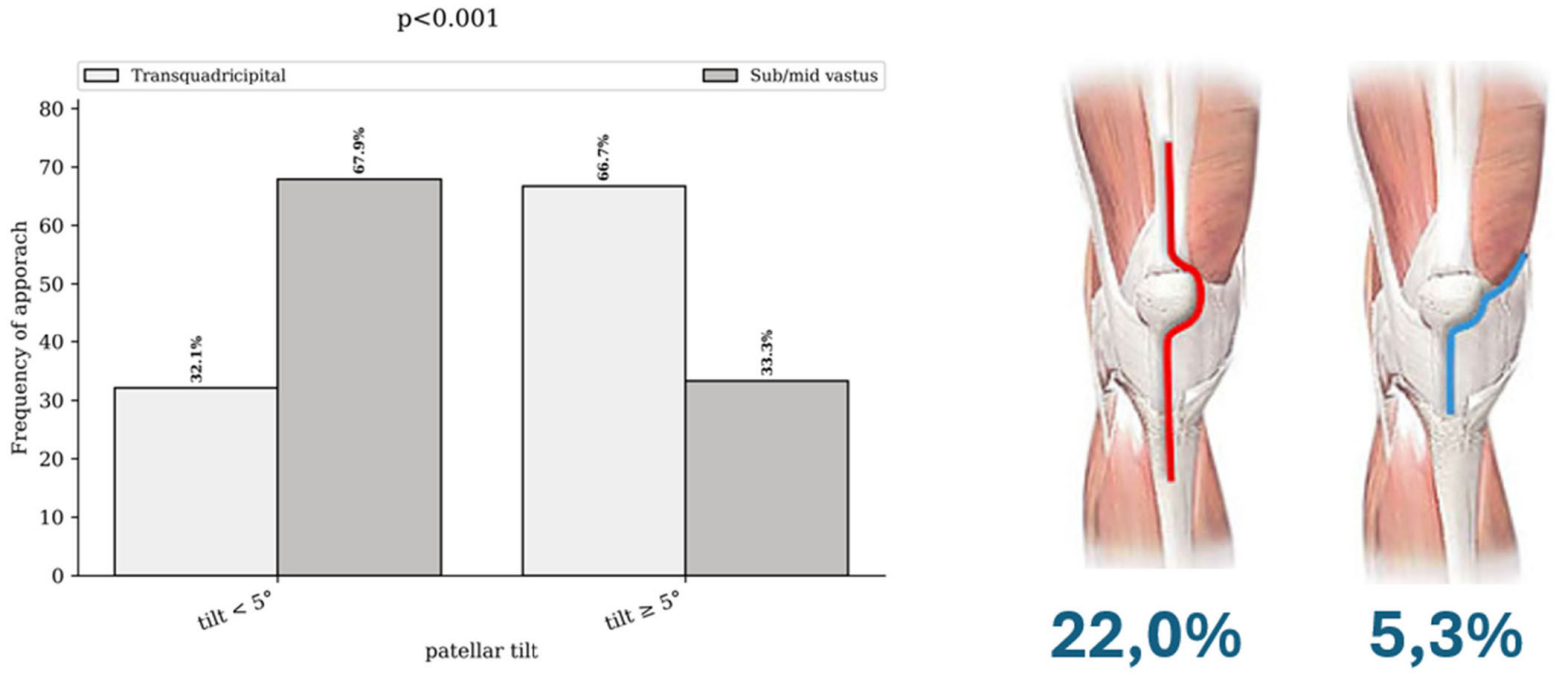
Incidence of postoperative tilt according to the approach.

**Figure 4:**
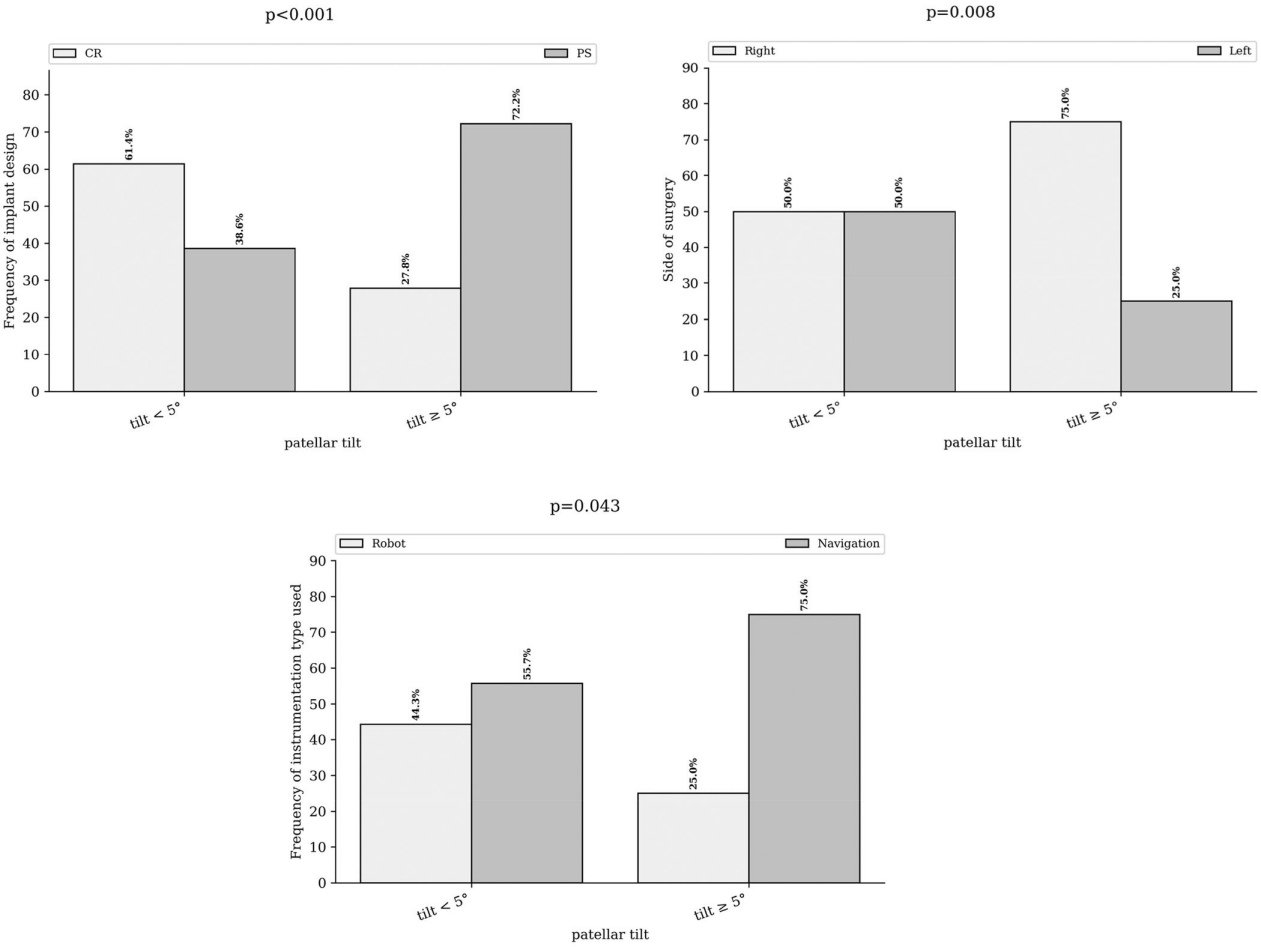
Postoperative tilt by implant design, surgical side and use of assistance.

The groups differed significantly in terms of surgical approach (*p* < 0.001), with an incidence of 5.3% in the subvastus approach group compared to 22.0% in the transquadricipital approach group (Figure 3). Significant differences were also observed regarding the operated side (*p* = 0.008), the use of navigation or robotic assistance (*p* = 0.043) and the implant design (*p* < 0.001) (Figure 4).

**Figure 5:**
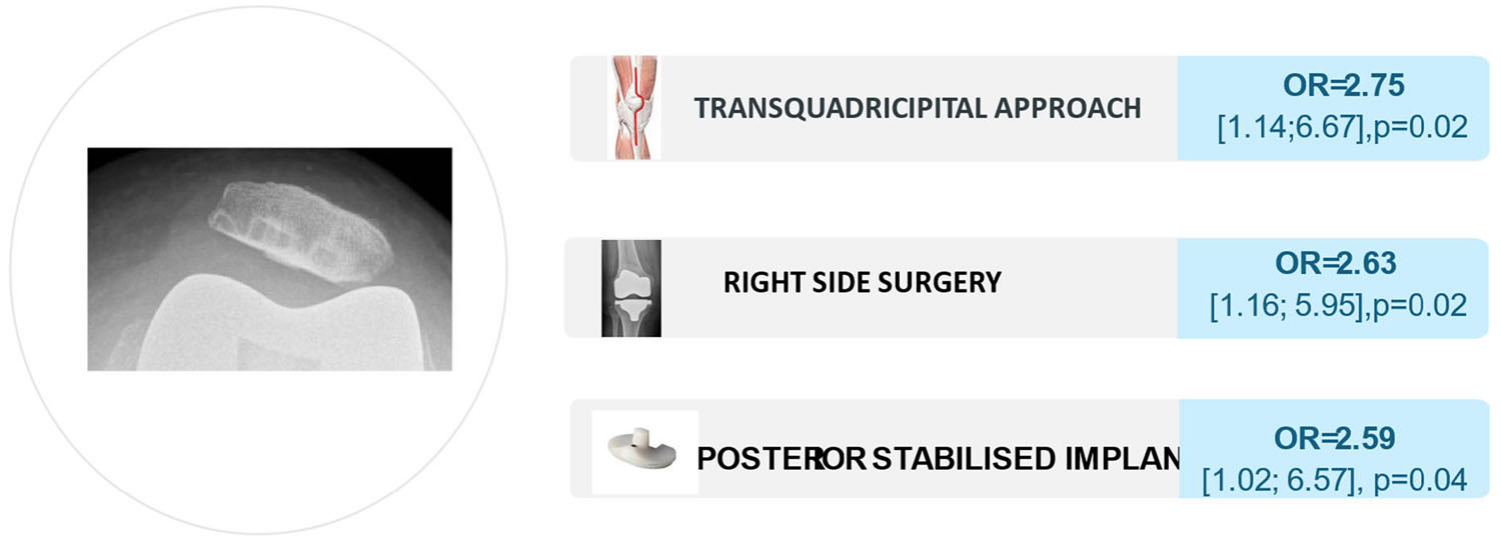
Factors independently and significantly associated with postoperative patellar tilt at 3 months.

Multivariate analysis identified the transquadricipital approach (odds ratio [OR] = 2.75 [1.14–6.67], *p* = 0.02), right‐sided procedures (OR = 2.63 [1.16–5.95], *p* = 0.02) and use of PS implants (OR = 2.59 [1.02–6.57], *p* = 0.04) as independent risk factors for patellar tilt (Figure 5).

During follow‐up, complications were observed in eight patients within the cohort: three infections (one requiring revision surgery, and two managed with a single debridement, antibiotics and implant retention), two cases of stiffness requiring manipulation under anesthesia, one femoral fracture following a fall, one case of clunk syndrome, and one patellofemoral dislocation. Notably, the patient who experienced the dislocation had a postoperative patellar tilt of 7 degrees.

## DISCUSSION

The main finding of this study is that personalized alignment does not increase, and likely reduces, the risk of patellar tilt, with a postoperative incidence of just 11.8%.

The relatively low incidence of postoperative patella tilt contrasts with the literature, where rates of up to 30% have been reported with mechanical alignment [19, 36]. These findings may be attributed to the ability of personalized alignment to more accurately restore the native trochlear anatomy. Rivière et al. reported that the femoral trochlear groove in kinematic alignment was positioned less in valgus and more medially compared to the mechanically aligned component, demonstrating greater similarity to the native trochlear morphology [40]. These features result in a patella that is captured early in flexion, promoting stability without overly constraining the tracking [40]. Nevertheless, these findings should be interpreted with consideration of recent ‘patella‐friendly’ implant designs, which may also contribute to improved trochlear morphology restoration [20, 28]. ‘Patella‐friendly’ refers to femoral implants with trochleae that improve stress distribution and patellar tracking [28]. These designs feature a deeper, proximally wider trochlear groove with lateral orientation and an elevated lateral facet, intended to replicate the morphology of a nondysplastic native trochlea [28]. The elevated lateral facet is essential in preventing lateral patellar subluxation or tilt [28].

The Attune implant used is this study is designed with an anatomical trochlear groove and a medialised patellar component, contrasting with the dome shape of previous implants [38]. To date, the only comparative study available reported a higher incidence of postoperative patellar tilt in patients who received the ATTUNE implant compared to those implanted with the PFC Sigma prosthesis [7]. This difference was attributed to the medialised design of the ATTUNE patellar component, which may induce a lateral tilting moment due to the off‐center pull of the quadriceps tendon. This finding underscores that postoperative patellar tilt cannot be attributed to implant design alone; rather, it is a multifactorial phenomenon. As described in the literature, intraoperative other factors such as lateral release [3] patellar resection thickness and asymmetry [14, 19], or patellar component positioning on the native patella [27] can also affect patellar kinematics.

At 3 months postoperatively, the transquadricipital approach emerged as an independent risk factor for patellar tilt. This supports existing literature showing that minimally invasive and vastus medialis–sparing approaches are associated with reduced patellar tilt and translation [18, 28]. Tilt tended to worsen postoperatively with the transquadricipital approach, with more lateral subluxation than with mid‐ and subvastus approaches [18, 28]. Additionally, lateral retinacular release was less commonly necessary when using the subvastus approach than with the transquadricipital approach [31].

Right‐sided procedures were identified as an associated factor for increased postoperative patellar tilt. In this study, all arthroplasties were performed with either navigation or robotic assistance for the femoral and tibial cuts, ensuring high precision and reproducibility. However, the patellar cut remains the only step not supported by navigated cutting guide and is performed manually using a conventional resection guide. This absence of technological assistance may introduce variability in the patellar resection, potentially influenced by the surgeon's handedness or ergonomic preferences when operating on one side versus the other.

This hypothesis aligns with previous reports suggesting that surgical ergonomics and dominant hand usage can influence patellar preparation accuracy [41]. However, it is important to note that no significant asymmetry in patellar bone resection was found between left and right sides in our cohort, despite the well‐documented impact of resection asymmetry on patellar tracking and instability [14].

Therefore, although the statistical significance of the right side in relation to patellar tilt is acknowledged, this finding should be interpreted with caution. It may represent incidental data rather than a true causal relationship.

Lastly, the use of PS implants has also been demonstrated as an associated factor. Studies already reported that posterior‐stabilized prosthesis had worst patellofemoral performance attributed to a less ‘patella‐friendly’ design characteristics [46].

The univariate analysis showed a significant difference in the incidence of postoperative tilt between the surgical assistance methods, with navigation being associated with a higher rate of tilt. However, this unexpected result was not confirmed in the multivariate analysis. Further research is needed to assess the potential lack of statistical power in this study. It should be noted that navigation‐assisted surgery involves placing guides on the femur and tibia, which may be less precise compared to using an articulating robotic arm.

### Limitations

First, while this was a retrospective study, the data analyzed were collected prospectively. Second, postoperative tilt was assessed at 3 months whereas other studies assess it at 1 year [19, 36]. At 3 months, postoperative hematoma can temporarily increase the tilt incidence and assess only early postoperative complications. Therefore, the follow‐up should be continued to have the same temporary assessment as reported in the literature. This study did not compare the results of conventional radiographs to CT scan. A direct correlation between the degree of patellar tilt and the external rotation of the femoral component was not established. The surgical approaches were performed by different pairs of surgeons—two performed all the transquadricipital approaches, while another two performed the subvastus approaches. This introduces a potential confounding factor, making it impossible to distinguish the specific effect of the surgical approach from the influence of individual surgeon technique or experience even if all the surgeons follow the same alignment strategy. Future studies should aim to control for this variable, ideally by having all approaches performed by the same group of surgeons or randomizing surgeons across techniques. Lastly, as this study is retrospective in nature, postoperative full weight‐bearing X‐rays were not routinely obtained. Consequently, postoperative CPAK and FKP could not be reported. This limits the ability to assess alignment changes.

Knowing that postoperative patellar tilt is correlated with femoropatellar complications, a potential strategy to improve clinical outcomes in routine practice could be the use of a personalized alignment approach that respects the patient's preoperative alignment, combined with cruciate‐retaining implants and a subvastus surgical approach. This combination may help preserve patellar kinematics and reduce postoperative maltracking. However, these encouraging findings need to be confirmed by a well‐controlled prospective study that evaluates both conventional radiographs and CT scans. It would also be a source of interest for further studies to link these radiological data to clinical outcomes with longer follow‐up periods.

## CONCLUSION

Using personalized alignment, a subvastus approach, and a cruciate‐retaining implant appears to reduce the risk of postoperative patellar tilt.

## AUTHOR CONTRIBUTIONS

**Alexandre Le Guen**: Designed the study; interpreted the data; wrote and reviewed the manuscript. **Simon Marmor:** Designed the study; reviewed the manuscript. **Vincent Le Strat:** Designed the study; reviewed the manuscript. **Antoine Mouton:** Designed the study; reviewed the manuscript. **Thomas Aubert**: Designed the study; contributed to the data analysis; supervised the study; edited and reviewed the manuscript. All authors have read the final manuscript and approved the manuscript to be published. All authors contributed to the creation of this study.

## CONFLICT OF INTEREST STATEMENT

Antoine Mouton serves as a consultant for Corin, Amplitude, ConMed and Johnson & Johnson, and receives royalties from ConMed. Simon Marmor serves as a consultant for Amplitude and Johnson & Johnson and receives royalties from Amplitude. Thomas Aubert is a consultant for Corin and Johnson & Johnson. The remaining authors declare no conflict of interest.

## ETHICS STATEMENT

Referenced under CNIL MR004 2225508 Has been submitted to the Ethic Committee for Clinical Research of Groupe Hospitalier Diaconesses Croix Saint‐Simon on 2025/04/09. This study has received a favorable opinion on the April 18, 2025. The committee certifies that the study is in accordance with the scientific principles generally accepted, the ethical standards and with the regulation in force. Informed consent was obtained from all patients.

## Data Availability

Data are available upon reasonable request.
